# Pixel-Wise Prediction of Glioblastoma Recurrence Using Mid-Treatment MRI and Deep Learning

**DOI:** 10.3390/cancers18213465

**Published:** 2026-10-27

**Authors:** Sara Rezvanjou, Laurentius Oscar Osapoetra, Daniel Moore-Palhares, Liam S. P. Lawrence, Angus Z. Lau, Arjun Sahgal, Gregory J. Czarnota

**Affiliations:** 1 Physical Sciences, Sunnybrook Research Institute, Toronto, ON M4N 3M5, Canada; 2 Department of Radiation Oncology, Odette Cancer Centre, Sunnybrook Health Sciences Centre, Toronto, ON M4N 3M5, Canada; 3 Department of Medical Biophysics, University of Toronto, Toronto, ON M5G 2C4, Canada; 4 Department of Radiation Oncology, University of Toronto, Toronto, ON M4N 3M5, Canada

**Keywords:** glioblastoma, apparent diffusion coefficient (ADC), recurrence-risk mapping, treatment resistance, deep learning, gross tumor volume (GTV), vision–language model

## Abstract

**Introduction**: Glioblastoma (GBM) commonly recurs close to the original gross tumor volume (GTV), suggesting treatment-resistant subregions may be present before radiologically visible progression. This study investigated whether deep learning on apparent diffusion coefficient (ADC) maps could generate pixel-wise probability maps of treatment-resistant regions within the GTV. **Methods**: The training/validation set included images of 64 in-house patients after radiation fraction 10 (Fx10) and 40 external patients before treatment used as an auxiliary cross-domain training cohort; the held-out test set included 16 in-house Fx10 patients. Resistant regions were defined as the overlap between the GTV from scans acquired at radiation fraction 10 and subsequent recurrence after finishing treatment. Models were trained on 2D GTV-cropped slices with patient-level separation to prevent data leakage. Single- and multimodal MRI inputs were compared, and ADC-based models (including baseline UNet, pretrained ResNet34, BiomedCLIP-assisted UNet, and a BLIP image-text UNet) were later evaluated. Performance was evaluated using conventional metrics, including ROC AUC, PR AUC, F1-score, recall, specificity, and precision. **Results**: ADC was the most informative single modality, achieving 69% and 80% for Receiver Operating Characteristic (ROC)- and Precision-Recall (PR)-Area Under the Curve (AUC), respectively. In the ADC-based architecture comparison, no model was consistently superior across all metrics. The baseline UNet achieved the highest accuracy, F1-score, and recall, whereas pretrained ResNet34 and BLIP-assisted models showed slightly higher ROC AUC than the baseline UNet (both 70%). Error analysis showed that performance decreased substantially for small or sparse resistant regions, and false-negative errors were enriched near resistant-region and GTV boundaries. **Conclusions**: ADC-based deep learning showed feasibility for pixel-wise spatial mapping of treatment-resistant glioblastoma regions within the GTV. A UNet trained from scratch provided the strongest sensitivity/F1 trade-off, whereas pretrained and VLM-assisted models provided only metric-specific benefits. Model comparisons showed that BiomedCLIP-assisted and BLIP image-text UNet models did not clearly improve fine spatial segmentation performance over the baseline UNet.

## Simple Summary

Glioblastoma can respond differently to treatment across different parts of the tumor, making it difficult to identify which regions are more likely to resis1t treatment. In this study, we compared different types of magnetic resonance imaging to determine which provided the most useful information for identifying resistant tumor regions early during treatment using deep learning. The deep learning models were trained to create maps showing where resistant tissue was more likely to be present within the original tumor area. Among the imaging types evaluated, diffusion-based imaging provided the best performance for distinguishing resistance from sensitive tissue, while combining it with other imaging types did not improve the results. The models performed less well when resistant regions were small or close to tumor boundaries. These findings may support future research toward more personalized treatment planning and monitoring.

## 1. Introduction

Glioblastoma (GBM) is the most common malignant primary brain tumor in adults and remains associated with poor survival despite multimodal treatment [1,2]. Current standard management typically includes maximal surgical resection followed by radiotherapy with concurrent and adjuvant temozolomide [2,3]. However, GBM is characterized by intratumoral heterogeneity, and treatment response is highly heterogeneous [4,5], varying not only across patients but also across different regions of the same tumor. Some tumor subregions respond to treatment, whereas others persist and later contribute to progression or recurrence. Early identification of resistant tumor regions is therefore clinically important because it could support timely treatment adaptation, more personalized management, and, in future applications, biologically guided dose painting and spatial recurrence risk mapping.

Imaging-based prediction of treatment response has received increasing attention in oncology [6,7]. Particularly, radiomics combined with machine learning has shown promise for capturing intratumoral heterogeneity and supporting treatment-response assessment in glioblastoma [7,8,9].

Among MRI-derived biomarkers, diffusion-weighted MRI has been recognized as a potential early surrogate marker of treatment response in brain tumors [10,11], and the apparent diffusion coefficient (ADC), obtained from diffusion-weighted imaging (DWI), is of special interest because it reflects tissue microstructure and cellularity [12,13,14,15]. More recent studies in glioblastoma have shown that ADC changes during chemoradiation may identify adversely prognostic tumor regions and distinguish gross tumor volume (GTV) areas that later recur from those that remain recurrence-free [11,16]. These findings support the biological relevance of ADC-based imaging for identifying non-responsive (treatment-resistant) tumor subregions.

Meanwhile, deep learning has increasingly been adopted in medical imaging because it can learn directly from image data without manual feature engineering. In neuro-oncology, deep learning methods have demonstrated strong performance in tumor detection, classification, and segmentation from MRI, particularly in benchmark settings such as BraTS and in robust segmentation frameworks such as nnUNet [17,18]. With growing interest in automated imaging pipelines to support clinical decision-making, identifying whether ADC provides useful information for predicting future glioblastoma recurrence regions is practically important. ADC is commonly acquired as part of brain tumor MRI protocols and is widely available on standard clinical scanners [19]. Therefore, if ADC-based models can reliably identify treatment-resistant subregions, this could motivate larger validation studies and support future efforts to standardize ADC acquisition, storage, and quantitative processing for automated recurrence-risk mapping. In addition, voxel-level and regional analyses have suggested that ADC may help predict where glioblastoma recurrence is likely to emerge [20]. Previous deep-learning studies of spatial recurrence prediction have used preoperative or pre-radiotherapy MRI and focused on peritumoral or other high-risk regions [21,22]. However, pixel-wise discrimination of resistant and sensitive tissue within the GTV using on-treatment MRI has received limited attention. Unlike the relatively coarse task of separating tumors from healthy brain, this problem requires the model to detect subtle intratumoral differences while learning from labels that are uncertain and highly imbalanced. As a result, models that perform well in conventional tumor segmentation may still struggle to identify biologically resistant subregions inside an already abnormal tumor volume. These challenges are amplified by the limited size of most clinically curated datasets.

In this study, we investigated whether deep learning models can distinguish treatment-resistant pixels from sensitive pixels within the glioblastoma GTV using MRI acquired during radiation treatment, with a particular focus on ADC-based imaging. We compared single-modality and multimodal MRI inputs, a conventional UNet model, pretrained segmentation backbones, and vision–language model (VLM)-assisted architectures to evaluate whether ADC and semantically richer image representations can support this fine-grained intratumoral prediction task. The mid-radiotherapy timepoint is expected to be a superior predictor of recurrence compared to pre-treatment timepoints because the response of the treatment-resistant and sensitive regions of the tumor may be detectable in the imaging. Because the in-house dataset was limited, we also evaluated whether adding a related but biologically non-equivalent cohort acquired at a different imaging time point could improve performance on the fixed in-house test set when used as auxiliary cross-domain training data.

We hypothesized that pixel-wise prediction of resistant tumor regions is feasible despite limited sample size, class imbalance, and subtle imaging differences between resistant and sensitive tissue. We further hypothesized that pretrained and VLM-derived features may provide complementary information to standard UNet models, while the segmentation network remains responsible for spatial localization and boundary refinement. Therefore, the aims of this study were: (1) to determine whether deep learning can generate spatial probability maps of resistant tumor regions within the GTV; (2) to compare ADC with other MRI input modalities for this task; (3) to evaluate whether conventional pretraining and VLM-assisted feature integration improve performance compared with a baseline UNet; and (4) to analyze how resistant-region burden and boundary-related uncertainty affect model errors.

## 2. Materials and Methods

### 2.1. Participant Selection

Initial training and test data used in this study came from clinical work [11] that provided a cohort of 80 patients with GBM treated at Sunnybrook Health Sciences Center, (Toronto, ON, Canada) that underwent longitudinal MRI from time of radiation planning (with available ADC, fluid-attenuated inversion recovery (FLAIR), and T1-weighted (T1w) modalities), imaging during treatment (at three time points of Fx0 (planning), Fx10 (radiation fraction10), Fx20 (radiation fraction 20), which Fx denotes number of fractions), and at one month after completing a 6-week course of chemoradiation (P1M) and later at time of recurrence (clinical follow up scans). Although planning and later on-treatment imaging were available, the present study focused on Fx10 because it represents an early treatment time point at which previous work in this cohort had already shown differences in ADC response between resistant and sensitive GTV regions [11]. The MRI was acquired by the Philips Ingenia system with a 1.5T magnetic field and the Philips Ingenia Elition X system with a 3.0 T magnetic field [23]. DWI (b-values: 0–1000 s/mm^2^) was acquired, and ADC maps were fitted voxel-wise using linear least-squares fitting to the log-signal decay versus b-value [11]. Structural alignment and anatomical references were maintained across all time points using T1-weighted and T2-FLAIR imaging. Recurrence was contoured at the earliest timepoint where magnetic resonance imaging (MRI) showed progression [11]. The gross tumor volume (GTV) was defined and delineated as the residual enhancing tumor and the surgical cavity. Delineation was done by radiation oncologists manually using the contrast-enhanced T1-weighted images [11] and validated by treatment group consensus. Image registration was performed to obtain the recurrence maps at each timepoint for analysis, utilizing the recurrence map at the time of progression [11]. The intersection of the GTV and recurrence was considered resistant to treatment and was labeled “1”. The rest of the GTV was considered sensitive to treatment and was assigned a label of “0”. This label definition was chosen to focus only on intratumoral resistance within the original treatment target, since previous research showed most treatment failures involved inside the GTV [11]. Therefore, recurrence outside the original GTV was not included in this study. This design also allowed the model to be evaluated on the more specific task of distinguishing resistant from sensitive tissue within the same abnormal tumor volume.

Per patient 2D slice-level images were used as model inputs to increase the number of training samples. Although this approach sacrifices inter-slice spatial information, it increases the effective dataset size for deep learning.

To further expand the training cohort, images from a publicly available longitudinal dataset [24] were incorporated. This data (including 40 patients) came from the Río Hortega University Hospital Glioblastoma (RHUH-GBM) dataset [24], which was obtained from The Cancer Imaging Archive (TCIA) repository. This publicly available collection provided preoperative, early postoperative, and recurrence multiparametric MRI scans with expert-validated segmentations. Both “in-house” and RHUH-GBM datasets contained ADC, FLAIR, and T1-weighted MRI and used the same definition of recurrence (the first image demonstrating recurrence after completion of chemotherapy). However, the baseline imaging time point differed between cohorts. In the “in-house” dataset, scans were acquired at radiation fraction 10 (Fx10), whereas the external dataset used scans acquired after surgery but before initiation of radiotherapy. Also, the RHUH-GBM dataset was acquired using three 1.5-T scanners from GE and Philips across two centers, with differences in image resolution and sequence parameters from our in-house dataset. DWI acquisition also differed, with the RHUH-GBM dataset using b-values of 0 and 1000 s/mm^2^, while the in-house protocol incorporated b-values across the 0–1000 s/mm^2^ range. These differences introduced additional acquisition-related heterogeneity into the combined training cohort. Therefore, the RHUH-GBM cohort was not treated as biologically similar to the in-house cohort. It was used as an auxiliary cross-domain training cohort to test whether increased training diversity could improve performance on the fixed in-house test set. This design was motivated by the limited size of the in-house cohort and by transfer-learning principles, where related source-domain data may improve learning when labeled target-domain data are limited.

For variables in which clinical or demographic information was unavailable in either dataset, the corresponding values were treated as missing data during demographic analysis and downstream applications.

### 2.2. Preprocessing

To reduce inter-scan variability, image sets underwent intensity standardization, scaling the normalized intensity range, and resampling to a uniform 1 mm isotropic grid using third-order B-spline interpolation; binary masks were resampled to the same grid with nearest neighbor interpolation. The resampled images were then normalized. To avoid background contamination, a brain mask was derived from the raw ADC images using non-background thresholding, morphological closing, and hole filling. GTV, recurrence, and cavity masks were constrained to this ADC-defined support; cavity voxels were removed from tumor masks, and recurrence was further restricted to lie within the cleaned GTV.

Slice selection was subsequently performed on the cleaned masks according to cohort-specific criteria. For patients with partial or no tumor recurrence, only slices with a gross tumor volume (GTV) area greater than 50 pixels were included. This threshold was chosen to reduce class imbalance by limiting the number of non-recurrence (sensitive) slices while retaining enough training data. In addition, very small tumor regions were excluded because they were considered unlikely to provide meaningful information for model training and could further exacerbate the class imbalance problem. For patients with complete tumor recurrence, all slices containing GTV were included in the analysis. The selected threshold was empirically determined based on the characteristics of the current dataset to balance data retention and class distribution.

For each retained slice, the image was cropped to the cleaned GTV bounding box, contrast was enhanced using Contrast Limited Adaptive Histogram Equalization (CLAHE), and all images and masks were resized to 256 × 256. The final binary target was defined within the GTV as resistant to treatment (labeled as 1) versus sensitive to treatment (labeled as 0) tissues.

### 2.3. Model Architecture

**Figure 1:**
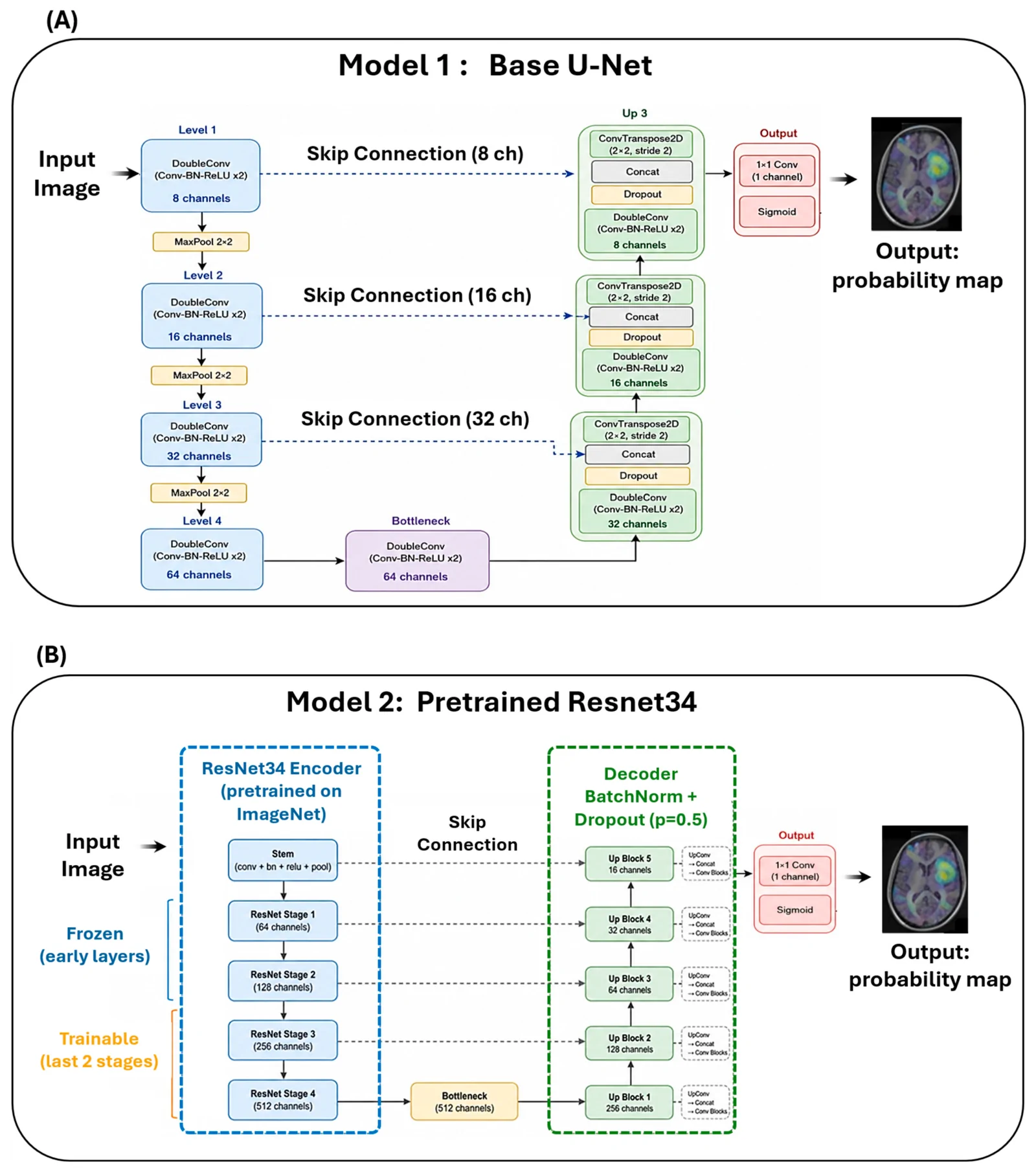

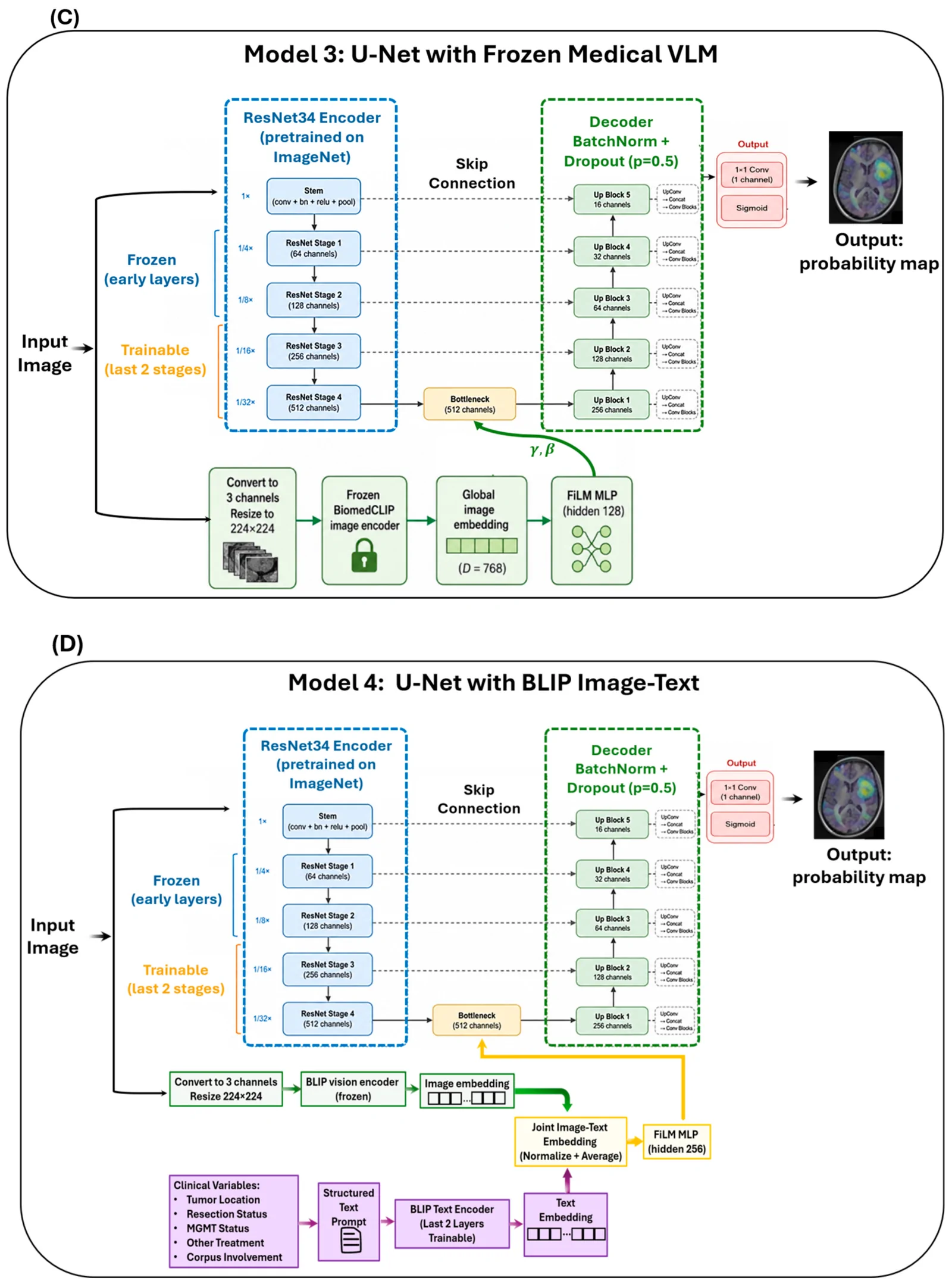
(**A**) Schematic of the 2D UNet backbone used for voxel-wise resistant region segmentation within the GTV. The network consists of a four-level encoder with channel widths of 8, 16, 32, and 64, followed by a symmetric decoder with transposed-convolution upsampling and skip connections. Each convolutional block includes convolution, batch normalization, and ReLU activation. The final 1 × 1 convolution generates a single-logit probability map for resistant tissue within the GTV. (**B**) Schematic of the pretrained UNet model using a pretrained ResNet34 encoder and a UNet decoder. Earlier encoder stages were frozen, while the final two encoder stages, decoder, and segmentation head were trainable. Decoder feature maps were progressively upsampled using channel widths of 256, 128, 64, 32, and 16, with batch normalization and dropout applied in the decoder. The output layer produced a logit probability map for resistant tissue within the GTV. (**C**) Schematic of the UNet model incorporating frozen medical vision–language model (VLM) features at the bottleneck. The ADC slice was processed by the UNet segmentation branch and, in parallel, by a frozen pre-trained medical vision–language model, BiomedCLIP (medical contrastive language image pretraining) image encoder, to extract a global image-level embedding. A small multilayer perceptron generated feature-wise linear modulation (FiLM) parameters, γ and β, which scaled and shifted the UNet bottleneck features before decoding. (**D**) Schematic of the Bootstrapping Language Image Pretraining (BLIP)-guided UNet model. The ADC slice was encoded by the UNet segmentation branch and by a frozen BLIP vision encoder, while selected clinical variables were converted into structured text prompts and encoded by the BLIP text encoder. Clinical text information, including tumor location (temporal, frontal, basal ganglia, thalamus, 3rd ventricle), resection status (STR, GTR, biopsy), MGMT status (methylated, unmethylated), additional treatments (lomustine, radiation, avastin, retreatment RT, resection, re-resection, dose-dense TMZ), and corpus callosum involvement, was transformed into structured text prompts and encoded using the BLIP text encoder. STR = Subtotal Resection; GTR = Gross Total Resection; MGMT = O^6^-methylguanine-DNA methyltransferase; TMZ = Temozolomide.

Firstly, to investigate which MRI modality is more informative for a deep learning model to discriminate between resistant and sensitive pixels, a simple 2D UNet was designed as the base model. This model used MRI modalities as a single input channel and consisted of an initial double-convolution block followed by an encoder with four resolution levels defined by channel widths of 8, 16, 32, and 64. Downsampling was performed by max-pooling followed by double convolutions, while the decoder used transposed convolutions for upsampling, skip-connection concatenation from the matching encoder levels. A final 1 × 1 convolution produced a single logit map. Batch normalization was also enabled throughout the network (Figure 1A). To investigate the impact of multimodal input, the previous model architecture was extended to a multimodal setting. In this regard, the same UNet framework was kept, and the input layer was updated to accept three image channels corresponding to ADC, FLAIR, and T1W+contrast, while preserving the same encoder–decoder architecture. All these models were trained from scratch.

To identify the most effective deep learning architecture for the selected input data, several model configurations were investigated. These models were chosen to evaluate the impact of different pretraining strategies, including models pretrained on natural image datasets (Pretrained ResNet34), models pretrained on medical imaging datasets (UNet with frozen medical VLM bottleneck fusion), and multimodal architectures incorporating both imaging and clinical data (UNet with BLIP image-text bottleneck fusion). This comparison enabled assessment of the contribution of domain-specific pretraining and clinical information to model performance.

#### 2.3.1. Pretrained ResNet34

In this model, a 2D UNet employed a pretrained ResNet34 encoder initialized with ImageNet weights, an encoder depth of 5, and a decoder with channel sizes of 256, 128, 64, 32, and 16. The decoder used batch normalization, and dropout was set to 0.50. To limit overfitting while still adapting the model to the target task, only the last two encoder stages were unfrozen, whereas the earlier encoder stages remained frozen; the decoder and segmentation head were fully trainable. Like the first model, the final layer produced a single logit map corresponding to the probability of resistant tissue within the GTV (Figure 1B).

#### 2.3.2. UNet with Frozen Medical VLM Bottleneck Fusion

To include semantically rich pretrained image representations while preserving the original UNet segmentation architecture, a frozen pretrained medical vision–language model, BiomedCLIP (medical contrastive language image pretraining) [25], was added as an auxiliary branch (Figure 1C). Specifically, each ADC slice was processed through the standard UNet pathway to generate the segmentation output. Simultaneously, the same ADC slice was replicated into three channels, resized to 224 × 224 pixels, and normalized before being passed to the frozen BiomedCLIP image encoder. The encoder extracted a global feature representation that captured high-level medical semantic information from the image. This feature vector was then passed through a small multi-layer perceptron (MLP), which generated Feature-wise Linear Modulation (FiLM) parameters (γ and β). These parameters were applied at the UNet bottleneck to modulate the feature maps and inject the semantic information learned by BiomedCLIP. The modulated features were subsequently processed by the decoder to produce the final segmentation mask. This design enabled the model to leverage pretrained medical image knowledge without altering the core segmentation structure.

In this model, instead of using a VLM as the main segmentation network, VLM-derived features were integrated into our best-performing UNet framework through a lightweight fusion strategy. This allows the model to benefit from high-level semantic information while preserving the strong spatial feature learning capabilities of the original segmentation network.

#### 2.3.3. UNet with BLIP Image-Text Bottleneck Fusion

This model used the same UNet segmentation backbone as the previous approach, with a pretrained ResNet34 encoder consisting of four encoder stages and matching decoder channel sizes. In addition to the ADC image, this model incorporated multimodal guidance using Bootstrapping Language Image Pretraining (BLIP) [26] at the bottleneck layer (Figure 1D). For the imaging branch, similar to the last model, each ADC slice was converted into a three-channel image, resized to 224 × 224 pixels, normalized, and processed by the BLIP vision encoder to generate an image embedding. Clinical text information, including tumor location (temporal, frontal, basal ganglia, thalamus, 3rd ventricle), resection status (STR, GTR, biopsy), MGMT status (methylated, unmethylated), additional treatments (lomustine, radiation, avastin, retreatment RT, resection, re-resection, dose-dense TMZ), and corpus callosum involvement, was transformed into structured text prompts and encoded using the BLIP text encoder. The image and text embeddings were normalized and combined to create a joint multimodal representation. This representation was then passed through a Feature-wise Linear Modulation (FiLM) module, which generated scaling and shifting parameters used to modulate the UNet bottleneck features. The modulated features were subsequently processed by the UNet decoder to produce the final binary segmentation map. To leverage pretrained knowledge while maintaining training stability, the BLIP vision encoder remained frozen throughout training, whereas the final layers of the text encoder were fine-tuned to better adapt the clinical information to the segmentation task.

### 2.4. Training Strategy

A patient-level random split was performed, with 80% of the main “in-house” dataset combined with the entire second dataset used for training and validation, while the remaining 20% of the main in-house dataset was reserved as an independent test set. The train/test separation was conducted at the patient level to ensure that no slices from the same patient appeared in both sets and to prevent data leakage. The RHUH-GBM cohort was added only to the training/validation set as auxiliary cross-domain data and was never included in the held-out test set. This design allowed us to evaluate whether related but non-equivalent external training data could improve performance on the intended in-house target domain while preserving patient-level separation and preventing data leakage.

All models were trained using patient-level, cohort-stratified 5-fold cross-validation. This approach ensured that all slices from a given patient remained within a single fold while maintaining a balanced distribution of cohorts across the training and validation sets. Model optimization was performed using the Adam optimizer with a learning rate of 2 × 10^−4^. The loss function consisted of a combination of binary cross-entropy with logits, focal loss and a soft Dice loss, supplemented with L1 and L2 regularization terms. To address class imbalance, a fold-specific positive-class weight was calculated from the training pixels. A default probability threshold of 0.50 was used for segmentation; however, during validation, this threshold was optimized to maximize balanced accuracy. To improve model generalization, geometric data augmentation was applied during training, including random rotations of up to 5° and translations of up to 8 pixels. Additional details regarding model training and hyperparameter settings are provided in Appendix A Table A1A,B.

### 2.5. Evaluation

Model evaluation was performed only within the GTV region. The primary performance metrics reported were accuracy, balanced accuracy, area under the receiver operating characteristic curve (ROC-AUC), area under the precision-recall curve (PR-AUC), F1 score, sensitivity, specificity, and precision. To estimate the uncertainty of test performance, patient-level bootstrap resampling with 500 iterations was performed to calculate 95% confidence intervals (CIs). All results presented in the tables correspond to performance on the independent test dataset, and the reported ± values represent the half-width of the 95% bootstrap confidence interval.

Composite performance summaries have been used in machine-learning studies as compact descriptive tools to compare models across multiple evaluation criteria [27,28]. In this study, we also reported a study-defined exploratory composite score only as a descriptive supplementary summary of metric trade-offs. The score was calculated as:

Overall Score = Accuracy + Balanced Accuracy + ROC-AUC + PR-AUC + Precision + Specificity + 2 × (F1 Score + Sensitivity)

The higher weights assigned to F1-score and sensitivity reflected the clinical motivation of reducing missed resistant pixels (FN) in future recurrence-risk mapping. However, this score is just an exploratory composite score, task-specific, and depends on the selected weighting scheme. Therefore, it was not used for statistical inference. This score and additional performance measures, including negative predictive value (NPV) and Brier score [29,30], were reported in Appendix A. Brier score was reported as a supplementary calibration-related metric to assess the agreement between predicted probabilities and binary resistant-region labels.

### 2.6. Error Analysis

To better understand model errors, subgroup and spatial error analyses were performed on the test set predictions. Slice-level Dice scores were grouped by the size of the ground truth (GT) resistant region within the GTV, and patient-level Dice scores were grouped by the number of slices containing resistant pixels. Conceptually, Dice measures spatial overlap between the predicted resistant region and the ground-truth resistant region (higher is better):

$$\mathrm{Dice}=\frac{2\mathrm{TP}}{2\mathrm{TP}+\mathrm{FP}+\mathrm{FN}}$$

These analyses assessed whether performance depended on the spatial burden of resistant tissue. Because multiple slices could come from the same patient, slice-level results were interpreted descriptively. We also analyzed false-positive and false-negative pixels by measuring their distance from the GT resistant region boundary and the GTV boundary. Errors were summarized across defined distance bands (from boundaries) to evaluate whether failures were mainly boundary-related or due to broader spatial mislocalization.

## 3. Results

### 3.1. Participant Characteristics

The training cohort (Table 1) included 104 patients (64 patients from the in-house dataset and 40 from the RHUH-GBM datasets), and the held-out test cohort included 16 patients from the in-house dataset. Age and sex distribution were not significantly different between the training and testing cohorts, and no statistically significant difference was observed for resection status or adjuvant TMZ use, although the training cohort showed some imbalance. In contrast, scanner magnet type showed a significant distributional difference, with a higher proportion of 3T scans in the test cohort. Because ADC values and image characteristics may vary between 1.5T and 3T acquisitions, scanner field strength was considered a potential source of test-set variability. An exploratory field-strength-stratified analysis was performed on the test set (Appendix A Table A2), which displays that, across models, 3T performance was lower for some metrics but comparable or higher for others. Because only five (31.2%) test patients were scanned at 3T, this analysis was interpreted descriptively and was not used for formal statistical comparison.

Additionally, overall survival showed a borderline difference (*p* = 0.05), with longer survival in the test cohort (860.25 ± 526.89 days) than in the training cohort (599.90 ± 385.53 days). The cohort-label distribution was broadly similar among patients with known recurrence-pattern labels. However, because the held-out test set was small and included only 3 patients with partial recurrence, pixel-level performance metrics may be influenced by recurrence-pattern distribution. Entire-recurrence and no-recurrence cases may contribute to patient-level class clustering and may not fully reflect the difficulty of fine spatial differentiation within partially recurrent tumors. Subgroup performance was additionally reported in Appendix A Table A3. More details about slice- and pixel-level distribution between train and test datasets are provided in Appendix A Table A4A,B.

### 3.2. Best Input Modality Selection

Table 2 compares model performance using different input modalities. To evaluate the impact of cross-domain expansion of training data, models trained using only the in-house cohort were compared to models trained using the combined in-house and RHUH-GBM set, while keeping the held-out test set fixed to the same in-house test patients. Among the single-modality models, the ADC-based model demonstrated the highest performance for both approaches. When only in-house data was used, the ADC-based model reached a balanced accuracy of 61 ± 7%, ROC AUC of 62 ± 8%, PR AUC of 73 ± 18%, and F1 score of 67 ± 11%. For the model trained on both in-house and auxiliary data, the ADC-based model showed higher performance compared to other modalities and compared to models trained only on in-house data, with a balanced accuracy of 63 ± 6%, ROC AUC of 69 ± 8%, PR AUC of 80 ± 14%, and F1 score of 72 ± 10%. However, Specificity decreased from 59% to 54%, indicating that the benefit was not uniform across all metrics. These results suggest that the RHUH-GBM cohort may have provided useful transferable information for the target test domain. In the extended training dataset (in-house + RHUH-GBM), T1W- and FLAIR-based models showed slightly lower but broadly similar performance except in terms of specificity. The combined T1W+FLAIR+ADC model achieved similar ROC AUC and PR AUC to the ADC-only model but showed lower specificity. Complementary metrics were reported in Appendix A Table A5. Overall, ADC appeared to be the most informative single modality according to all metrics and in both approaches, while adding T1W and FLAIR did not clearly improve segmentation performance. Also, the ADC model showed more consistent precision and recall, which is very important in the case of medical use since false positives and negatives can have critical consequences.

### 3.3. Models’ Performance

Table 3 summarizes the performance of the ADC-based deep learning architectures for both in-house and cross-domain expansion of training data. Comparing these two approaches displays a similar trend of increasing performance for models trained on extended datasets. In the extended training approach, the baseline UNet achieved the highest accuracy, F1-score, and recall, with values of 65 ± 7, 72 ± 10, and 71 ± 10, respectively. The pretrained ResNet34 and the UNet with BLIP image-text bottleneck fusion showed slightly higher ROC AUC than the baseline UNet, both reaching 70 ± 9 and 70 ± 8, respectively, compared with 69 ± 8 for the baseline model. The pretrained ResNet34 model also achieved the highest PR AUC numerically, although the difference was small compared with the other architectures.

**Figure 2:**
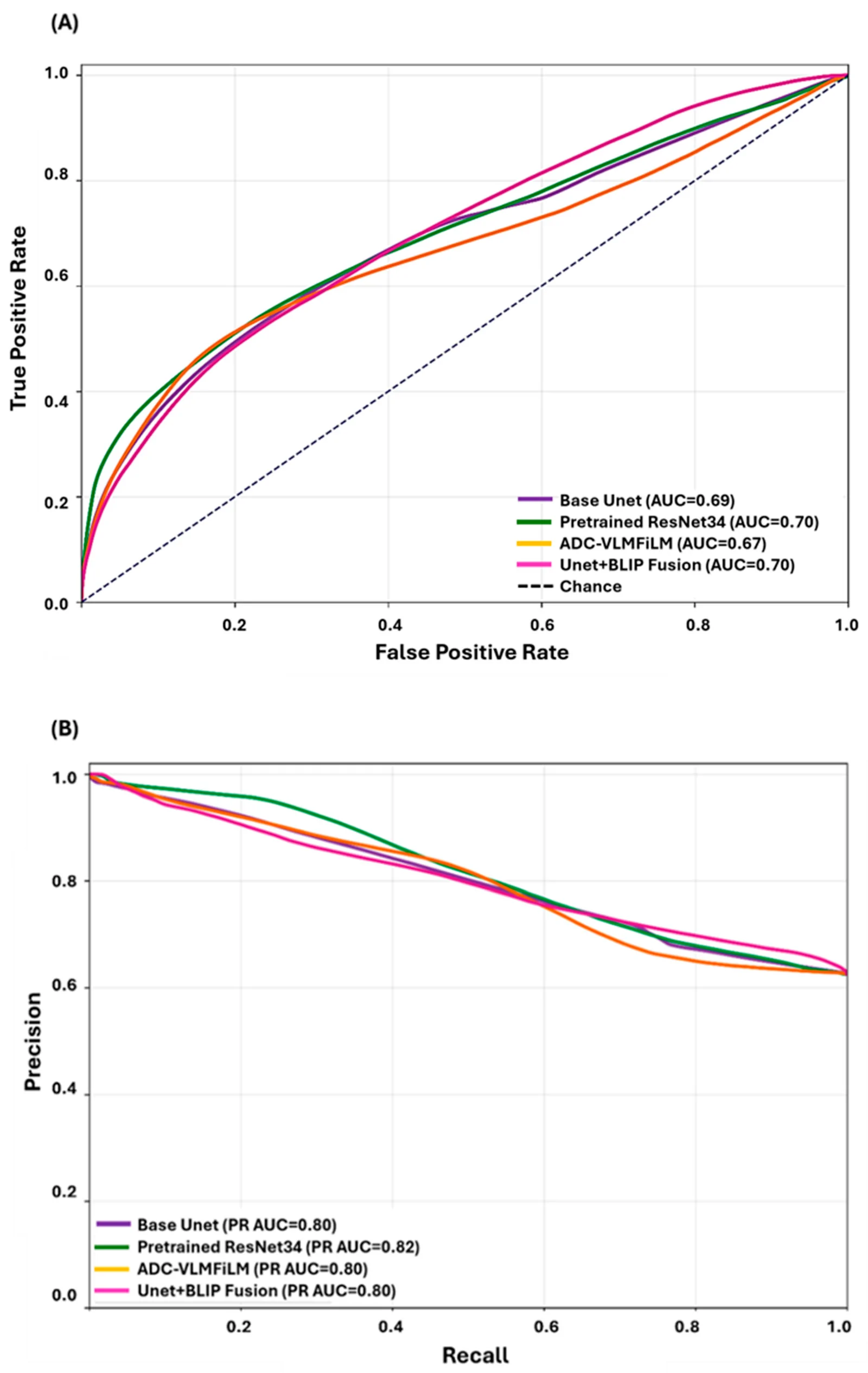
(**A**) Test-set ROC and (**B**) precision-recall performance of the models. Receiver operating characteristic and precision-recall curves on the test set. Curves were computed using voxel-wise predictions restricted to the GTV region. Between-model AUC differences were assessed using paired patient-level bootstrap resampling, and no model showed a robust AUC difference compared with UNet.

The UNet model with frozen medical VLM showed the highest balanced accuracy, specificity, and precision, with values of 65 ± 6, 74 ± 10, and 78 ± 13, respectively. However, this improvement in specificity and precision was accompanied by a lower recall and F1-score, suggesting a more conservative classification behavior with reduced sensitivity to resistant pixels. In contrast, the baseline UNet was more sensitive, but with lower specificity, indicating a greater tendency toward false-positive resistant-pixel classification. The baseline UNet showed the strongest sensitivity/F1 trade-off, achieving the highest recall and F1-score among the ADC-based models. The pretrained ResNet34 and BLIP-assisted models showed slightly higher ROC AUC, whereas the frozen medical VLM achieved higher specificity and precision. As shown in Table 3, incorporating the auxiliary training data improved performance on the fixed primary test set. Because the auxiliary dataset used a different baseline imaging point relative to the first chemotherapy dose, this improvement was interpreted as reflecting the benefit of increased training diversity. Complementary metrics were reported in Appendix A Table A6. Figure 2 shows the corresponding ROC and precision-recall curves for the ADC-based architecture. Between-model AUC differences were assessed using paired patient-level bootstrap resampling, and no robust AUC difference was observed. However, the small number of independent test patients limited the power to detect robust differences between architectures. Patient-level 3D Dice showed substantial variability across the test patients with GTV recurrence (Appendix A Table A7). When Dice was calculated separately for each patient across the whole retained GTV, performance varied across patients and models. This variability indicates that aggregate pixel-level and subgroup-level results may mask patient-level heterogeneity.

### 3.4. Error Analysis

**Figure 3:**
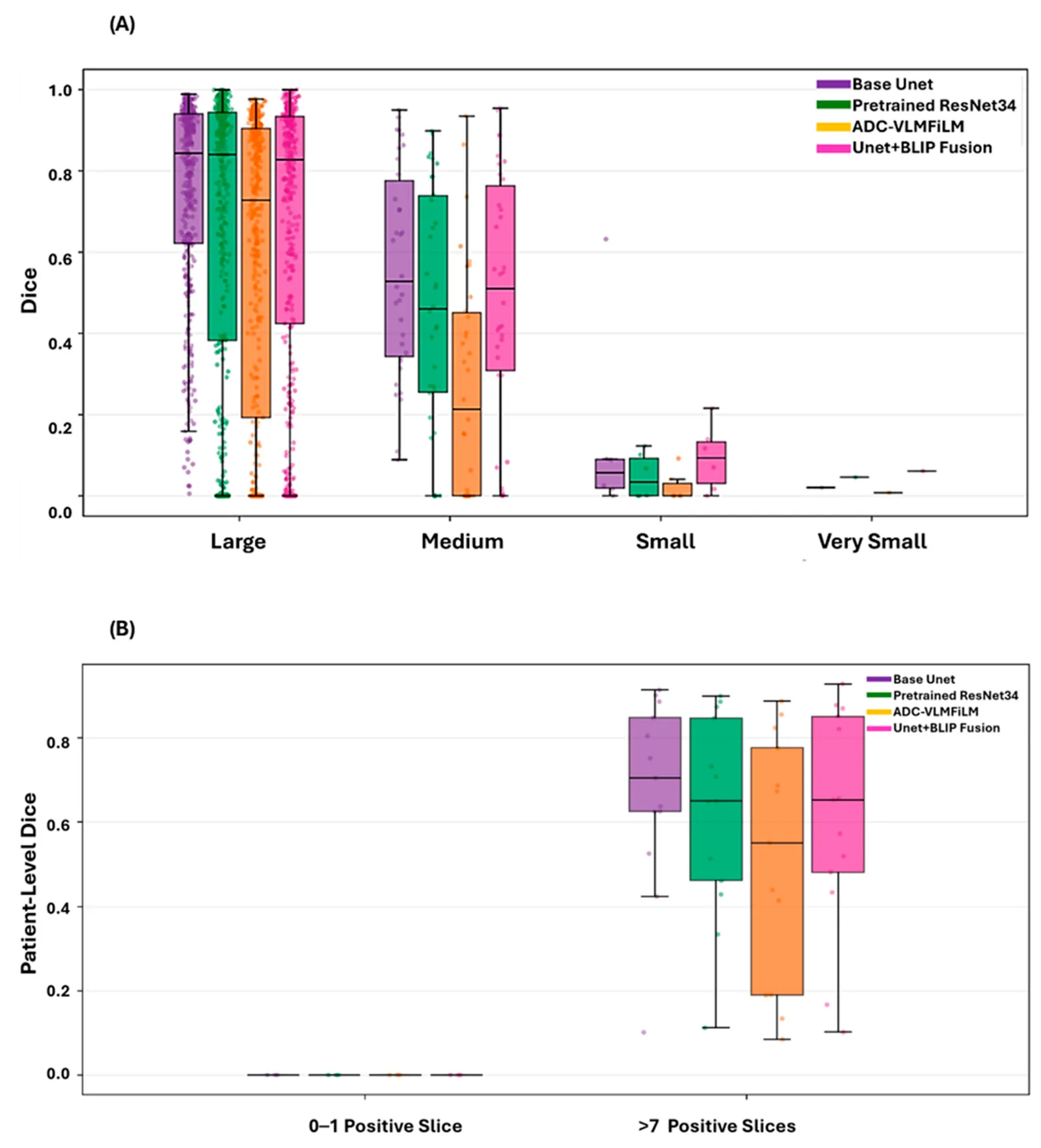
Effect of resistant-region burden and spatial error distribution on segmentation performance. (**A**) Slice-level Dice coefficient stratified by ground-truth resistant-region size, defined as the number of pixels with a positive label within the GTV. Bins were defined as: very small = 1–499 pixels, small = 500–2499 pixels, medium = 2500–9999 pixels, and large = ≥10,000 pixels. Slices with zero resistant pixels were excluded from Dice stratification. (**B**) Patient-level Dice coefficient stratified by the number of slices containing resistant tissue. Patient-level Dice was computed by pooling true-positive, false-positive, and false-negative pixels across all test slices for each patient.

Error analysis was performed to assess the influence of resistant-region burden and spatial error distribution on model performance (on models trained on the extended dataset). As shown in Figure 3A, slice-level Dice scores were strongly related to the size of the ground-truth resistant region within the GTV. All models achieved higher Dice values for slices with large resistant regions, whereas performance decreased for medium, small, and very small regions. This trend indicates that segmentation performance was more reliable when the resistant region occupied a larger spatial extent within the slice. For small and very small regions, Dice values were low across architectures, reflecting the greater sensitivity of pixel-level segmentation metrics to small absolute errors when the target region is limited.

At the patient level, Figure 3B showed a similar dependence on resistant-region burden. Patients with only 0–1 positive slices had Dice values close to zero across all models, while patients with more than seven positive slices showed higher and more variable but meaningful Dice values. This suggests that model performance was reduced when resistant pixels were sparse across the tumor volume and improved when resistant tissue was present across a larger number of slices. Therefore, low-burden cases represent a challenging condition for ADC-based resistant-region segmentation.

**Figure 4:**
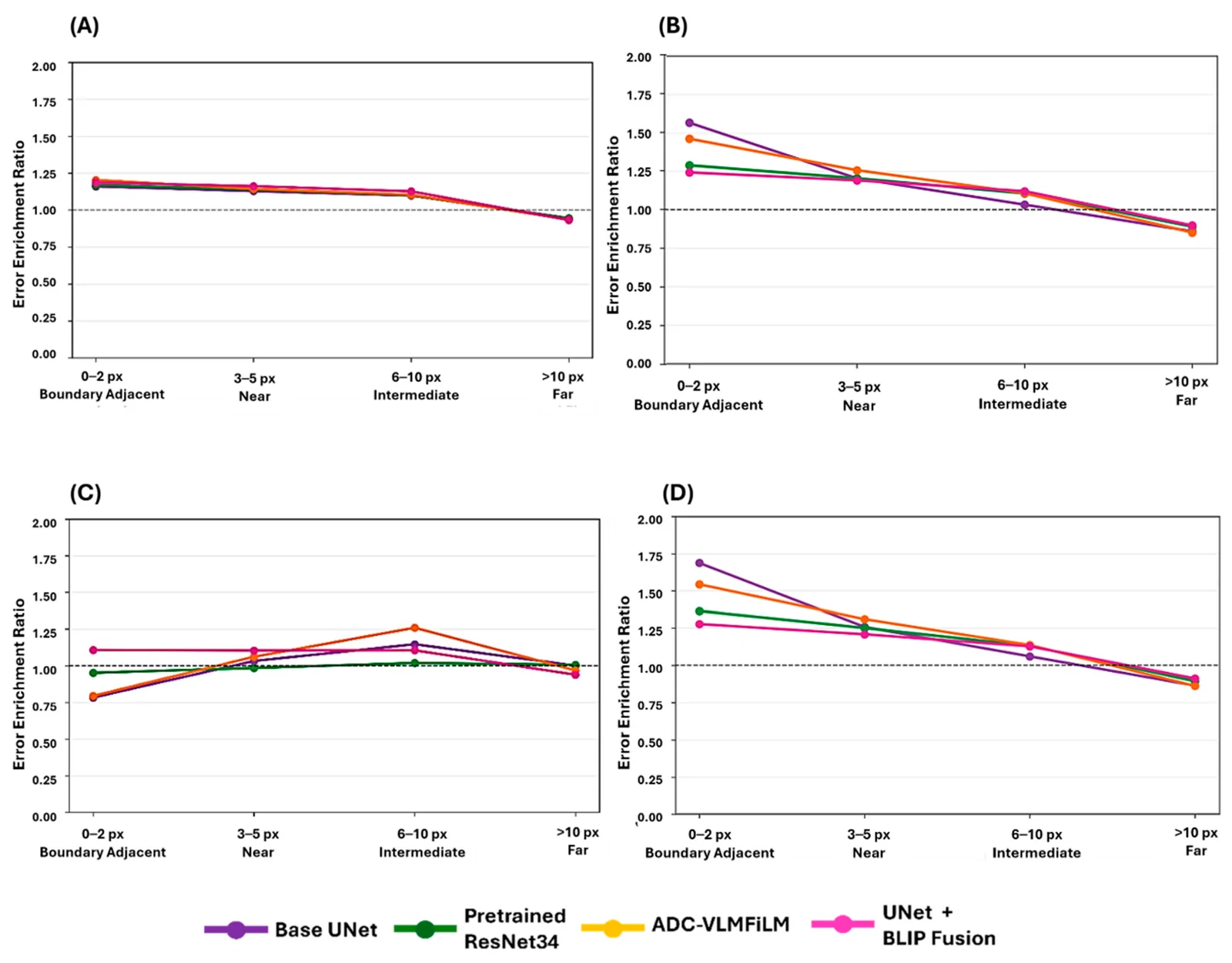
Boundary-distance enrichment of prediction errors, area-adjusted enrichment to distance from the ground-truth resistant-region boundary and the GTV boundary. (**A**) False positives near resistance boundary, (**B**) false negative near resistance boundary, (**C**) false positive near GTV boundary, (**D**) false negative near GTV boundary. Enrichment ratio was calculated as the fraction of error pixels in each distance band divided by the fraction of eligible ROI pixels in the same band. Values greater than 1 indicate that errors occurred more frequently than expected by available area, and enrichment lower than 1 shows errors are less common there than expected in that band. For FP, eligible area is GT-sensitive ROI pixels, while for FN, eligible area is GT-resistant pixels.

**Figure 5:**
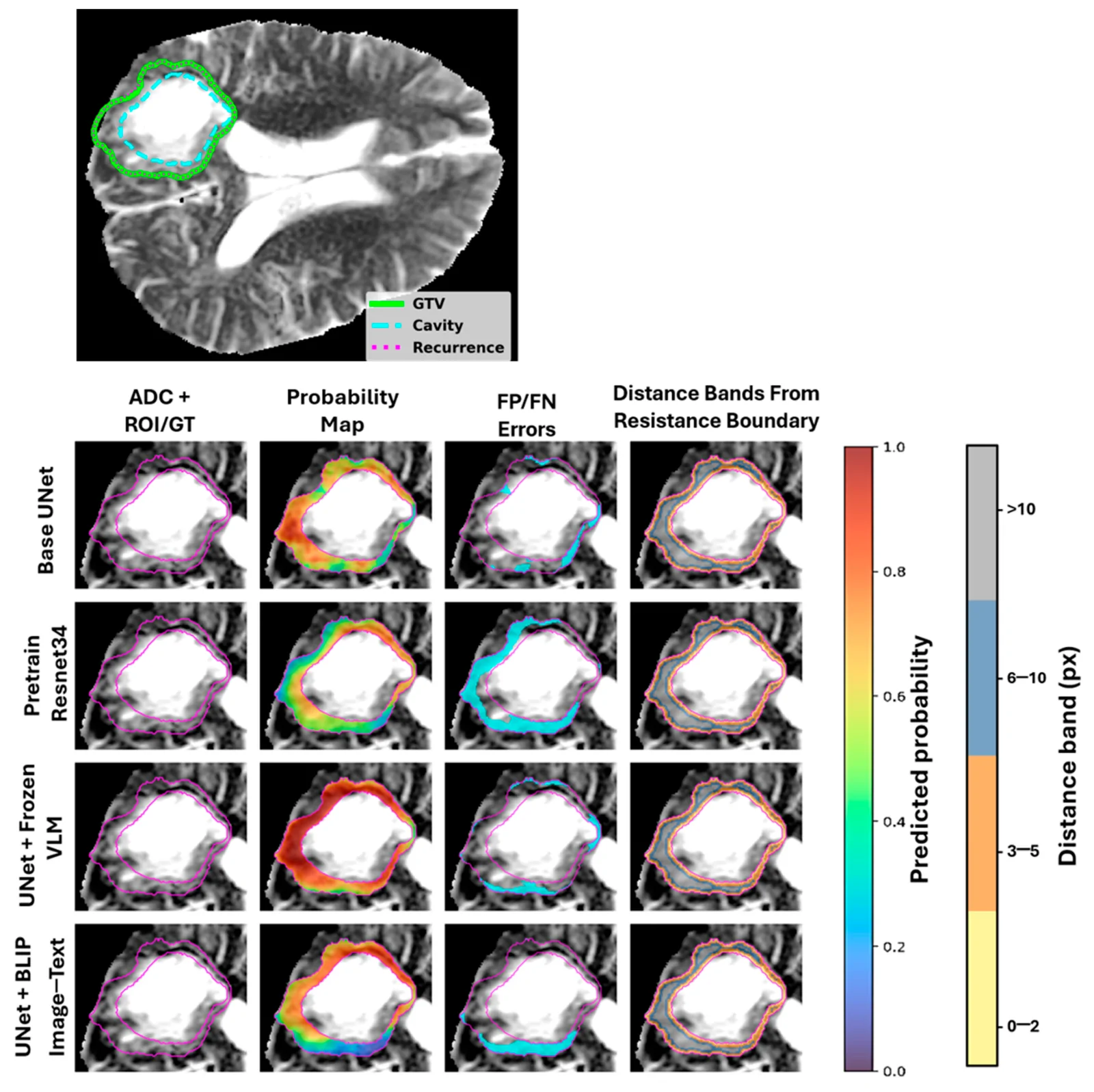
Representative test-set slices illustrating spatial patterns of false-positive and false-negative predictions across models, highlighting where prediction errors were concentrated near the resistant region or GTV boundaries. Error overlays are shown relative to predefined boundary-distance bands, with yellow indicating boundary-adjacent pixels (0–2 px), orange near-boundary pixels (3–5 px), blue intermediate-distance pixels (6–10 px), and gray distant pixels (>10 px). A representative slice was selected to highlight a case where prediction errors were concentrated near the resistant-region or GTV boundaries.

**Figure A1:**
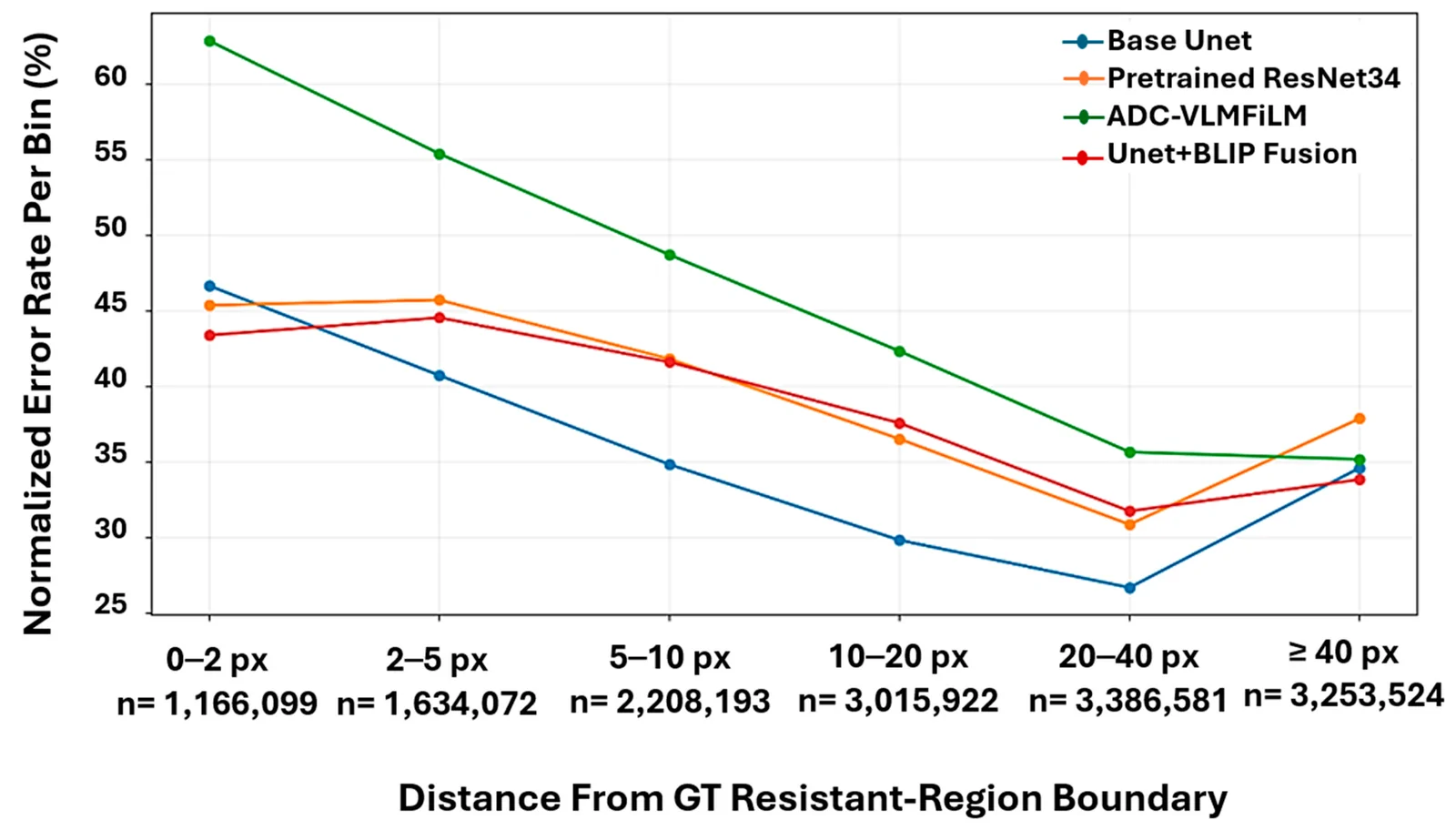
Normalized prediction error as a function of distance from the ground-truth resistant-region boundary. Normalized model error rate calculated across distance bands from the ground-truth (GT) resistant-region boundary. Error rate was calculated as the number of false positive plus false negative pixels divided by the total number of eligible GTV pixels within each distance band. Higher values near the boundary indicate boundary-localized errors, whereas persistent errors at larger distances suggest broader spatial mislocalization. The number of GTV pixels contributing to each distance band is shown on the x-axis.

In Figure 4, false-negative errors showed clearer error enrichment near the resistant-region and GTV boundaries, especially for the baseline UNet, while false-positive errors were less strongly boundary-localized. The clearest boundary-related failure mode is false-negative errors are enriched near the resistant-region and GTV boundaries, especially for Base UNet. But false positives are not strongly boundary-localized. Normalized prediction error as a function of distance from the resistant-region boundary was shown in Appendix A Figure A1. A representative example in Figure 5 visually supported these patterns by showing the relationship between false positive/false negative pixels and the distance bands from the resistant-region boundary.

## 4. Discussion

This study investigated the ability of deep learning models to generate spatial maps of treatment-resistant regions within the glioblastoma gross tumor volume (GTV), with a particular focus on ADC-based MRI. The main finding was that ADC was the most informative modality for pixel-wise discrimination between resistant and sensitive tissue within the GTV. Compared with T1W and FLAIR, the ADC-based model performed better across most metrics. The better performance of ADC compared to T1W and FLAIR is consistent with the biological role of diffusion MRI in reflecting tissue microstructure and cellularity [10]. The use of Fx10 imaging, acquired early during treatment, was also supported by a previous study in the same cohort, which showed that differences in ADC response between resistant and sensitive GTV regions were already apparent at this early on-treatment time point [11]. This is also consistent with previous studies in high-grade glioma showing that diffusion-related changes measured during treatment can provide early information on therapeutic response and patient outcome [31,32]. Together, these findings suggest that treatment-resistant tumor regions may exhibit early diffusion-related changes after treatment initiation that are not clearly visible on conventional anatomical MRI. Combining ADC with T1W and FLAIR did not clearly improve performance. This may be due to several factors, including the limited sample size, increased input complexity, and the possibility that non-ADC sequences add information that is less directly related to the resistant-tissue label.

The architectural comparison showed that different model modifications improved different aspects of performance, but no single architecture consistently outperformed the others across all evaluation metrics, and after accounting for patient-level clustering, small numerical AUC differences between architectures were not robust, suggesting that no architecture showed clear superiority in this limited test cohort. Based on the exploratory composite score defined in this study, the baseline UNet achieved the highest rank while maintaining the smallest model size (Appendix A Table A6); however, this composite score is study-defined and weight-dependent and was only used as a parameter to rank models in this study application and not considered as the main metric to compare models. Also, because the held-out test cohort is small, model ranking should be interpreted cautiously. Therefore, the architecture comparison was interpreted as metric-specific trade-offs, not definitive superiority of one model. Although the pretrained ResNet34 and BLIP-assisted models achieved slightly higher ROC AUC values, these improvements did not consistently translate into better sensitivity, specificity, or precision. Moreover, these models required approximately 180 times as many trainable parameters as the baseline UNet, substantially increasing computational training cost. In contrast, the medical VLM-assisted model achieved the highest specificity and precision, indicating improved false-positive control, but at the expense of lower sensitivity. Notably, this model required approximately one-third as many trainable parameters as the ResNet34 and BLIP-assisted models, while remaining more computationally demanding than the baseline UNet. These findings suggest that VLM-assisted and BLIP-assisted models may be insufficient for fine intratumoral boundary-level prediction unless further adapted to the target segmentation task. Additionally, the Brier scores suggested that calibration of the raw probability outputs was limited and varied across the models (Appendix A Table A6). The Base UNet showed the most favorable Brier score, whereas the VLM-assisted and BLIP-assisted models showed weaker calibration-related performance. Therefore, the predicted probability maps should be interpreted as relative spatial risk maps. Future work should include dedicated calibration analysis using validation-set calibration, expected calibration error, and Brier Skill Score.

This trade-off is important for the intended application. If the goal is future recurrence-risk mapping or biologically guided treatment adaptation, missed resistant pixels may be more clinically concerning than conservative overprediction. In this context, the conventional UNet trained from scratch showed stronger recall and F1-score. These results suggested that semantically richer pretrained representations could change model behavior, but they did not necessarily outperform a base model on this fine-grained, segmentation-based pixel-level task.

Vision–language models, including BLIP and CLIP-based frameworks, have recently attracted attention in medical imaging because they can encode richer image-level representations than conventional convolutional backbones. However, in this study, transfer learning from pretraining and general vision–language representations provided only modest benefit. One likely explanation is a mismatch between generic pretrained features and the subtle local patterns needed to distinguish resistant from non-resistant tissue within the same tumor. Frozen VLM features may capture high-level semantic information, but they may not preserve the local spatial detail required for pixel-wise segmentation and boundary refinement. The baseline UNet may therefore remain better suited to local spatial pattern extraction in this setting, although it is still limited by small training datasets, class imbalance, and noisy labels. This may reflect the difficulty of distinguishing resistant from non-resistant tissue at the pixel level, as well as the fact that pretrained models, even when trained on medical or general imaging data, may not be directly optimized for this highly specific intratumoral prediction task. These findings support cautious use of advanced deep learning architectures for highly specific pixel-level prediction tasks, particularly when added computational complexity does not produce clear performance improvement.

The patient-level 3D Dice analysis (Appendix A Table A7) confirmed substantial inter-patient variability. Although some patients had high Dice scores, performance was not consistent across the cohort, particularly in partial-recurrence cases. These findings support cautious interpretation of aggregate pixel-level and subgroup-level metrics and highlight the need for larger validation cohorts enriched for partial-recurrence cases. Additionally, results demonstrated that incorporating the auxiliary training cohort improved performance on the fixed primary test set, despite differences in treatment timing, with in-house images acquired during concurrent chemoradiation (Fx10) and RHUH images acquired early postoperatively, within 72 h of surgery [24]; therefore, the RHUH-GBM cohort was incorporated as an auxiliary cross-domain training cohort, not as biologically equivalent data. ADC values are known to change during and after treatment as a result of treatment-related biological processes such as necrosis, inflammation, and cellular swelling [13]. Training on images acquired at different treatment stages exposes the model to physiologically plausible intensity variations, encouraging it to learn stable anatomical and pathological characteristics instead of overfitting to treatment-stage-specific signal changes. Therefore, the two cohorts should not be interpreted as representing the same biological imaging state. However, both cohorts included glioblastoma MRI with ADC, FLAIR, and T1-weighted imaging and recurrence-based spatial labels, so the RHUH-GBM data provided related but heterogeneous source-domain examples. Also, this additional temporal variability increases sample diversity, potentially improves robustness to variations in image acquisition and scanner characteristics, and may have helped the model learn transferable imaging representations useful for the target task by encouraging the learning of more general features while reducing overfitting to institution-specific or treatment-stage-specific characteristics. So, this temporal difference was intentionally retained as a domain expansion strategy rather than treated as a source of bias. However, this should not be interpreted as evidence of biological equivalence between pre-radiotherapy and on-treatment imaging. Instead, the results support a cross-domain training interpretation: related heterogeneous data may improve robustness in a limited-data setting, but larger multi-institutional Fx10 datasets and formal harmonization or domain-adaptation strategies are needed to confirm generalizability in future studies.

The error analysis showed that model performance was strongly affected by resistant-region burden. Slice-level Dice scores were higher when resistant regions were large, but decreased considerably for medium, small, and very small regions. A similar pattern was observed at the patient level: patients with only a few positive slices had Dice values close to zero, whereas patients with a larger number of positive slices showed more meaningful segmentation performance. These findings indicate that sparse resistant disease is a major limitation for pixel-level resistance mapping.

The boundary analysis suggested that model errors arose from more than one source. False-negative errors were more concentrated near the resistant region and GTV boundaries (Figure 4), suggesting that uncertainty in boundary definition and spatial registration may have contributed to missed resistant pixels. In contrast, false-positive errors were less consistently localized near boundaries and, in some cases, occurred farther from the ground-truth resistant region, indicating broader spatial mislocalization.

Previous studies have shown that ADC can provide biologically relevant information about tumor cellularity, treatment response, and recurrence risk in glioblastoma [11]. Radiomics studies have also suggested that ADC-derived texture and intensity features may help identify tumor subregions that later progress or recur [33]. However, the previous study mainly relied on handcrafted radiomic features and voxel-level machine learning rather than end-to-end deep learning segmentation of resistant tissue within the GTV. The present study extends that by evaluating whether deep-learning models can directly learn spatial patterns associated with treatment-resistant tumor regions from MRI. This is a more challenging task than conventional tumor segmentation because the model is not separating tumor tissues from normal brain tissues. Instead, it must distinguish subtle biological differences within an already abnormal tumor volume. These resistant regions may be small, spatially sparse, and affected by uncertainties related to recurrence registration and GTV delineation. Therefore, the observed performance should be interpreted as feasibility evidence for intratumoral resistance mapping, rather than as proof of clinical readiness.

A key contribution of this study is its focus on pixel-wise resistance mapping within the GTV, instead of whole-tumor segmentation or patient-level recurrence classification, which has been less extensively investigated. This distinction is important because treatment-resistant tissue may involve only part of the original tumor volume. Preserving this spatial information may support future recurrence-risk mapping and biologically guided treatment adaptation. This study also compared different MRI input strategies and model architectures under the same prediction task. In addition, the error analysis helped clarify when and why the models failed. Together, these analyses provide a more detailed assessment of the feasibility and limitations of deep learning-based intratumoral resistance mapping.

This study has several limitations. First, the available dataset size was limited for a pixel-wise intratumoral prediction task, particularly given the biological heterogeneity of glioblastoma and the strong imbalance between resistant and sensitive pixels. The held-out test cohort was also relatively small, with only three partial GTV recurrence patients. The recurrence-pattern distribution of the held-out test set is an important limitation. Only three test patients had partial GTV recurrence, which is the most informative scenario for evaluating true intratumoral spatial differentiation. In contrast, entire-GTV recurrence and no-GTV recurrence cases may simplify some pixel-level metrics by introducing patient-level class clustering. The subgroup analysis (Appendix A Table A3) showed that performance in partial-recurrence cases was modest and lower than in the entire-recurrence subgroup, confirming that overall Dice and PR-AUC should be interpreted cautiously. Therefore, the current results should be considered preliminary feasibility evidence, and future validation should include larger cohorts enriched for partial-recurrence cases and independent external testing.

In addition, the auxiliary RHUH-GBM cohort differed from the in-house cohort in treatment timing, scanner characteristics, and acquisition protocol. Although this cohort improved several test-set metrics when used as auxiliary training data, treatment-stage differences may introduce biological and imaging-domain shifts. Thus, these results are considered preliminary evidence for cross-domain training and require validation in larger multi-institutional Fx10 datasets. Also, Scanner field strength differed between the training and test cohorts, with a higher proportion of 3T scans in the test set. Exploratory stratified analysis (Appendix A Table A2) suggested that performance differences were not uniform across metrics; however, PR AUC and precision were lower in the 3T subgroup for the UNet model. Because the 3T subgroup included only five patients, these differences may reflect field strength, acquisition protocol, recurrence-pattern distribution, or patient-level variability. Therefore, field-strength-stratified analysis was limited in this study. Future validation should include larger scanner-balanced or scanner-harmonized cohorts.

Second, the models were trained using 2D slices. This approach increased the number of training samples but did not explicitly capture through-plane spatial continuity across neighboring slices. Because resistant tumor regions are three-dimensional structures, future 3D models may better characterize volumetric patterns within the GTV.

Third, the error analysis showed that performance was strongly influenced by resistant-region burden, with poorer segmentation for small and sparse target regions. Boundary-related uncertainty also contributed to false-negative errors. Because resistant labels were derived from registered recurrence maps, boundary and label uncertainty may arise from deformable registration, recurrence contouring, and GTV delineation. Together, these findings suggest that future models may benefit from uncertainty-aware training, boundary-aware loss functions, or multi-stage designs that separately address localization and fine segmentation.

In addition, the VLM-based models were evaluated in a limited setting. The present findings do not exclude their potential value but suggest that frozen or weakly adapted VLM features may be insufficient for this fine pixel-level task. Task-specific fine-tuning, larger medical imaging datasets, and more careful integration with segmentation networks may be required before VLM-based approaches provide clear benefit. Finally, in this study, resistant tissue was defined only within the original GTV, so recurrence outside the original GTV was not modeled in this study, and the model should be interpreted as mapping resistant subregions within the original GTV, not as predicting all future recurrence.

Clinically, the results of this study suggest that ADC-based deep learning may be useful for generating preliminary spatial probability maps of treatment-resistant tumor regions. However, it is important to emphasize that the poorer performance of the investigated models for small and sparse resistant regions is an important limitation for future clinical applications, such as treatment adaptation or dose-painting strategies. Therefore, the present maps should not be interpreted as ready for treatment planning or clinical use, particularly for detecting small resistant subregions.

Future studies should evaluate these models in larger, multi-institutional cohorts and with independent external validation. Larger datasets would allow more reliable assessment of model robustness and clarify whether pretrained models from natural-image, medical-image, or vision–language domains provide complementary value for resistance mapping. Incorporating neighboring slices or full 3D context may also improve spatial consistency and localization. Another useful direction is to combine handcrafted radiomics features with deep learning representations, since radiomics may provide stable texture descriptors while deep learning can learn hierarchical spatial patterns directly from MRI.

## 5. Conclusions

Overall, these findings support ADC as a promising candidate for future automated recurrence-risk mapping in glioblastoma. Given the limited dataset size, class imbalance, and lack of independent external validation, the results of this study should be interpreted as preliminary feasibility evidence. This work provides a meaningful starting point for further investigation of ADC-based deep learning as a tool for spatially characterizing high-risk tumor subregions. Because ADC is widely available and already included in many brain tumor MRI protocols, the present findings motivate further development of standardized ADC-based imaging pipelines for recurrence-risk mapping. With larger multi-institutional datasets, improved spatial modeling, and uncertainty-aware validation, ADC-based resistance maps may eventually support research on biologically guided treatment adaptation, dose-painting strategies, and closer imaging follow-up of high-risk tumor regions. However, reliable detection of small and sparse resistant regions remains necessary before this approach can be considered for treatment adaptation or dose-painting applications.

## Figures and Tables

**Table 1:** Demographic and clinical characteristics of the training and test cohorts. Continuous variables are reported as mean ± standard deviation, and categorical variables are reported as number (%). *p*-values compare the training and test cohorts. GTR = gross total resection; NTR = near-total resection; STR = subtotal resection; TMZ = temozolomide; IDH = isocitrate dehydrogenase; WT = wild type.

Demographic Variable	Train (N = 104)	Test (N = 16)	*p*-Value
Age	55.4 ± 12.8	52.4 ± 14.3	0.544
Sex			0.258
Female	37 (35.6%)	3 (18.8%)	
Male	67 (64.4%)	13 (81.2%)	
Resection status			0.074
Biopsy	10 (9.6%)	3 (18.8%)	
GTR	47 (45.2%)	4 (25.0%)	
NTR	13 (12.5%)	0 (0.0%)	
STR	34 (32.7%)	9 (56.2%)	
Adjuvant TMZ			0.059
No	24 (23.1%)	7 (43.7%)	
Yes	74 (71.2%)	7 (43.7%)	
Unknown	6 (5.7%)	2 (12.5%)	
Overall survival (days)	599.90 ± 385.53	860.25 ± 526.89	0.050
IDH status			
Mutant	6 (5.8%)	1 (6.2%)	
WT	98 (94.2%)	15 (93.8%)	
Magnet Type			0.021
1.5T	95 (91.3%)	11 (68.8%)	
3T	9 (8.7%)	5 (31.2%)	
COHORT			
A—Partial	12 (18.8%)	3 (18.8%)	
B—No GTV Recurrence	13 (20.3%)	3 (18.8%)	
C—Entire GTV Recurrence	39 (60.9%)	10 (62.5%)	
D—Unknown	40 (38.5%))	0 (0%)	

**Table 2:** Comparison of model performance using different input modalities. Initial models trained only on in-house cohort (N = 64). Models with * show training on the total training dataset (N = 104) including both cohorts (in-house and RHUH-GBM). All reported metrics were calculated on the same separate in-house test dataset (N = 16). Acc = accuracy; Bal Acc = balanced accuracy; ROC AUC = receiver operating characteristic area under the curve; PR AUC = precision-recall area under the curve. The ± values represent the half-width of the 95% confidence interval estimated using patient-level bootstrap resampling with 500 iterations.

Input Modality	Acc	Bal Acc	ROC AUC	PR AUC	F1 Score	Recall	Specificity	Precision
T1W	55 ± 06	56 ± 07	58 ± 08	72 ± 18	59 ± 11	52 ± 09	60 ± 09	68 ± 20
FLAIR	56 ± 06	57 ± 07	59 ± 07	70 ± 18	61 ± 11	55 ± 09	58 ± 09	69 ± 20
ADC	61 ± 07	61 ± 07	62 ± 08	73 ± 18	67 ± 11	62 ± 09	59 ± 10	72 ± 18
T1W+FLAIR+ADC	58 ± 04	60 ± 05	63 ± 07	74 ± 18	61 ± 08	53 ± 06	66 ± 06	72 ± 19
T1W *	58 ± 10	52 ± 04	57 ± 07	70 ± 18	70 ± 12	78 ± 06	26 ± 06	64 ± 18
FLAIR *	60 ± 07	58 ± 05	62 ± 07	74 ± 16	68 ± 12	67 ± 09	49 ± 09	69 ± 18
ADC *	65 ± 07	63 ± 06	69 ± 08	80 ± 14	72 ± 10	71 ± 10	54 ± 10	72 ± 15
T1W+FLAIR+ADC *	62 ± 09	58 ± 05	64 ± 09	77 ± 17	71 ± 12	74 ± 08	42 ± 08	68 ± 18

Metrics Definitions: Accuracy = $\frac{\mathrm{TP}+\mathrm{TN}}{\mathrm{TP}+\mathrm{FN}+\mathrm{TN}+\mathrm{FP}}$, balanced Accuracy = $\frac{\left(\mathrm{Recall}+\mathrm{Specificity}\right)}{2}$, F1-Score = $\frac{2\times \mathrm{Precision}\times \mathrm{Recall}}{\mathrm{Precision}+\mathrm{Recall}}$, Recall = $\frac{\mathrm{TP}}{\mathrm{TP}+\mathrm{FN}}$, specificity = $\frac{\mathrm{TN}}{\mathrm{TN}+\mathrm{FP}}$, precision = $\frac{\mathrm{TP}}{\mathrm{TP}+\mathrm{FP}}$, where TP = True Positives, FN = False Negatives, TN = True Negatives, and FP = False Positives.

**Table 3:** Comparison of ADC-based deep learning model architectures. Initial models trained only on in-house cohort (N = 64). Models with * show training on the total training dataset (N = 104) including both cohorts (in-house and RHUH-GBM). All reported metrics are calculated on the same separate in-house test dataset (N = 16). Bal Acc = balanced accuracy; ROC AUC = receiver operating characteristic area under the curve; PR AUC = precision–recall area under the curve; VLM = vision–language model; BLIP = Bootstrapping Language-Image Pre-training. The ± values represent the half-width of the 95% confidence interval estimated using patient-level bootstrap resampling with 500 iterations.

Model	Acc	Bal-Acc	ROC AUC	PR AUC	F1-Score	Recall	Specificity	Precision
UNet	61 ± 07	61 ± 07	62 ± 08	73 ± 18	67 ± 11	62 ± 09	59 ± 10	72 ± 18
Pretrained ResNet34	61 ± 08	59 ± 07	64 ± 11	76 ± 17	68 ± 12	67 ± 12	50 ± 11	69 ± 18
UNet with frozen medical VLM	59 ± 08	59 ± 08	63 ± 10	75 ± 17	65 ± 13	60 ± 12	58 ± 11	70 ± 18
UNet *	65 ± 07	63 ± 06	69 ± 08	80 ± 14	72 ± 10	71 ± 10	54 ± 10	72 ± 15
Pretrained ResNet34 *	64 ± 07	63 ± 08	70 ± 09	81 ± 10	70 ± 10	66 ± 12	61 ± 13	74 ± 12
UNet with frozen medical VLM *	63 ± 08	65 ± 06	67 ± 09	80 ± 14	65 ± 12	56 ± 14	74 ± 10	78 ± 13
UNet with BLIP image-text *^,^**	64 ± 08	63 ± 07	70 ± 08	80 ± 12	70 ± 11	66 ± 12	61 ± 09	74 ± 14

** Clinical text information, including tumor location (temporal, frontal, basal ganglia, thalamus, 3rd ventricle), resection status (STR, GTR, biopsy), MGMT status (methylated, unmethylated), additional treatments (lomustine, radiation, avastin, retreatment RT, resection, re-resection, dose-dense TMZ), and corpus callosum involvement. STR = subtotal resection; GTR = gross total resection; MGMT = O^6^-methylguanine-DNA methyltransferase; TMZ = Temozolomide.

## Data Availability

The in-house datasets used and/or analyzed during the current study are available from the corresponding author on reasonable request.

## Abbreviations

The following abbreviations are used in this manuscript:

GBM	Glioblastoma
DWI	Diffusion-Weighted Imaging
ADC	Apparent Diffusion Coefficient
GTV	Gross Tumor Volume
FLAIR	Fluid-Attenuated Inversion Recovery
T1-W	T1-weighted
VLM	Vision–Language Models
BLIP	Bootstrapping Language Image Pretraining
CLIP	Contrastive Language Image Pretraining
STR	Subtotal Resection
GTR	Gross Total Resection
MGMT	O^6^-methylguanine-DNA methyltransferase
TMZ	Temozolomide

## Appendix A

**Table A1:** Key architecture and training parameters for (**A**): “UNet with frozen medical VLM” model. (**B**): “UNet with BLIP image-text” model.

Category	Setting
(**A**)	
Segmentation backbone	UNet
Encoder	ResNet-34
Encoder initialization	ImageNet pretrained
Auxiliary pretrained model	BiomedCLIP-PubMedBERT vision encoder
VLM input size	224 × 224 pixels
VLM branch	Frozen
Fusion strategy	FiLM-style bottleneck modulation
Fusion MLP hidden size	128
Fusion dropout	0.10
FiLM scaling factors	γ scale = 0.10; β scale = 0.10
UNet fine-tuning	Last 2 encoder stages unfrozen; decoder and segmentation head trainable
Batch size	8
Early stopping patience	8 epochs
Optimizer	AdamW
Learning rate	1 × 10^−4^
Weight decay	1 × 10^−5^
L1 regularization	λ = 1 × 10^−5^
Loss function	BCE + focal loss + soft dice
Dice loss weight	0.30
Focal loss weight	0.25
Focal loss parameters	γ = 2.0; α = 0.25
Augmentation probability	0.30
Rotation range	±5°
Translation range	±8 pixels
Initial threshold	0.50
Threshold tuning range	0.05 to 0.95, step = 0.01
(**B**)	
Segmentation backbone	UNet
Encoder	ResNet-34
Encoder initialization	ImageNet pretrained
Auxiliary pretrained model	BLIP image-captioning base
BLIP image input size	224 × 224 pixels
Text input	Structured prompt from selected clinical variables
Text variables	Tumor location, resection status, MGMT status, other treatments, corpus involvement
Maximum text length	64 tokens
Fusion strategy	Joint image-text embedding with FiLM-style bottleneck modulation
Fusion MLP hidden size	256
Fusion dropout	0.10
FiLM scaling factors	γ scale = 0.10; β scale = 0.10
BLIP vision branch	Frozen
BLIP text branch	Partially fine-tuned; last 2 text layers unfrozen
U-Net fine-tuning	Last 2 encoder stages unfrozen; decoder and segmentation head trainable
Batch size	10
Early stopping patience	10 epochs
Optimizer	AdamW
Learning rate	1 × 10^−4^
Weight decay	1 × 10^−5^
L1 regularization	λ = 1 × 10^−5^
Loss function	BCE + focal loss + soft dice
BCE loss weight	0.60
Focal loss weight	0.10
Dice loss weight	0.30
Focal loss parameters	γ = 2.0; α = 0.25
Augmentation probability	0.30
Rotation range	±5°
Translation range	±8 pixels
Initial threshold	0.50
Threshold tuning range	0.05 to 0.95, step = 0.01

**Table A2:** Exploratory test-set performance stratified by MRI field strength. Performance was evaluated separately for 1.5T and 3T test cases. The 3T subgroup showed lower PR AUC and precision for the UNet model, while specificity was slightly higher. Across models, the direction of the 1.5T–3T difference varied by metric. These findings suggest that scanner field strength may contribute to test-set variability, but the small number of 3T patients prevents a reliable conclusion about scanner-specific performance. * shows training on the total training dataset (N = 104) including both cohorts (in-house and RHUH-GBM). N = number of patients; Bal Acc = balanced accuracy; ROC AUC = receiver operating characteristic area under the curve; PR AUC = precision–recall area under the curve.

Model	Field Strength	N	Acc	Bal Acc	ROC AUC	PR AUC	Recall	Specificity	Precision
UNet *	1.5T	11	62 ± 9	60 ± 8	62 ± 11	82 ± 16	63 ± 11	57 ± 13	81 ± 16
	3T	5	59 ± 9	58 ± 10	58 ± 12	42 ± 16	56 ± 12	61 ± 12	40 ± 16
	1.5T, 3T	16	65 ± 7	63 ± 6	69 ± 8	80 ± 14	71 ± 10	54 ± 10	72 ± 15
Pretrained ResNet34 *	1.5T	11	63 ± 11	56 ± 6	64 ± 10	86 ± 12	72 ± 12	40 ± 11	78 ± 16
	3T	5	68 ± 10	64 ± 7	68 ± 10	57 ± 22	59 ± 3	69 ± 14	46 ± 41
	1.5T, 3T	16	64 ± 7	63 ± 8	70 ± 9	81 ± 10	66 ± 12	61 ± 13	74 ± 12
UNet with frozen medical VLM *	1.5T	11	60 ± 11	61 ± 8	65 ± 11	85 ± 13	57 ± 17	65 ± 18	83 ± 13
	3T	5	71 ± 15	64 ± 5	61 ± 9	50 ± 28	45 ± 8	83 ± 4	55 ± 43
	1.5T, 3T	16	63 ± 8	65 ± 6	67 ± 9	80 ± 14	56 ± 14	74 ± 10	78 ± 13

**Table A3:** Recurrence-pattern subgroup performance on the held-out test set. Three-dimensional Dice was computed by pooling true-positive, false-positive, and false-negative pixels across all retained GTV slices for each patient subgroup. The no-GTV recurrence subgroup was not included because it contains no positive resistant pixels; therefore, Dice, ROC AUC, PR AUC, recall, and precision are not directly meaningful for this subgroup. Recurrence-pattern subgroup analysis showed lower performance in partial-recurrence cases than in entire-GTV recurrence cases. This confirms that overall pixel-level Dice and PR AUC are influenced by recurrence-pattern distribution and should be interpreted cautiously. * Indicates training on the total training dataset (N = 104), including both cohorts (in-house and RHUH-GBM). N = number of patients; Bal Acc = balanced accuracy; ROC AUC = receiver operating characteristic area under the curve; PR AUC = precision–recall area under the curve.

Sub-Groups	Model	N	3D Dice	Acc	Bal Acc	ROC AUC	PR AUC	Recall	Specificity	Precision
Partial Recurrence	UNet *	3	0.56	54	55	55	54	65	46	50
	Pretrained ResNet34 *	3	0.52	52	52	54	52	58	46	47
	UNet with frozen medical VLM *	3	0.55	60	59	62	57	54	64	55
	UNet with BLIP image-text *	3	0.55	57	57	59	52	59	55	51
Entire GTV Recurrence	UNet *	10	0.81	69	58	65	90	73	44	90
	Pretrained ResNet34 *	10	0.77	65	59	64	93	67	51	91
	UNet with frozen medical VLM *	10	0.70	58	63	67	94	56	69	93
	UNet with BLIP image-text *	10	0.77	65	59	63	93	67	50	91

**Table A4:** (**A**) Cohort, slice, and voxel-level label distribution of training and testing datasets. The training/validation included the in-house Sunnybrook cohort and the additional RHUH-GBM cohort, while the held-out test included only in-house Sunnybrook patients. (**B**) Cohort, slice, and voxel-level label distribution of training/validation dataset (Sunnybrook and RHUH-GBM cohorts). GTV pixels = total pixels inside gross tumor volume; resistant pixels = the overlap between the GTV and recurrence map; sensitive pixels = GTV-only tissue within GTV; positive slices = slices containing at least one resistant pixel; positive pixel ratio = resistant pixels divided by total GTV pixels.

Split	Cohort	Patient, N	Slices, N	Positive Slices, N (%)	Negative Slices, N (%)	GTV Pixels, N	Resistance Pixels, N	Sensitive Pixels, N	Positive Pixel Ratio
(**A**)									
Train	Partial Recurrence	12	359	186 (51.8)	173 (48.2)	12,865,886	3,076,366	9,789,520	23.91%
	No GTV Recurrence	13	453	56 (12.4)	397 (87.6)	16,894,871	1,773,128	15,121,743	10.50%
	Entire GTV Recurrence	39	1343	1343 (100.0)	0 (0.0)	50,325,282	42,485,005	7,840,277	84.42%
	Unknown	40	1693	35 (89.7)	1003 (59.1)	56,442,588	10,516,075	45,926,513	18.63%
Test	Partial Recurrence	3	114	83 (72.8)	31 (27.2)	4,532,646	2,017,436	2,515,210	44.51%
	No GTV Recurrence	3	81	0 (0.0)	81 (100.0)	3,364,601	0	3,364,601	00.0%
	Entire GTV Recurrence	10	359	359 (100.0)	0 (0.0)	11,522,270	10,133,483	1,388,787	87.95%
(**B**)									
Train 1	Sunnybrook Hospital Cohort	64	2155	54 (84.4%)	1585 (73.5%)	80,086,039	47,334,499	32,751,540	59.10%
Train 2	RHUH-GBM Cohort	40	1698	35 (89.7%)	1003 (59.1%)	56,442,588	10,516,075	45,926,513	18.63%
Total	----	104	3853	89 (86.4%)	2588 (67.2%)	136,528,627	57,850,574	78,678,053	42.37%

**Table A5:** Complementary performance metrics for comparing different input modalities. Initial models trained only on in-house cohort (N = 64). Models with * show training on the total training dataset (N = 104) including both cohorts (in-house and RHUH-GBM). All reported metrics were calculated on the same separate in-house test dataset (N = 16). NPV = negative predictive value = $\frac{\mathrm{TN}}{\mathrm{TN}+\mathrm{FN}}$ (TN = True Negatives, FN = False Negatives); overall score was defined as different weights assigned to metrics according to their importance in clinical application = Accuracy + Balanced Accuracy + ROC AUC + PR AUC + Precision + Specificity + 2 × (F1-Score + Sensitivity). Brier’s score was used to evaluate the accuracy of the probabilistic predictions, where lower scores indicate better predictive performance [29,30]. The Brier scores range from 0 to 1; where 0 is perfect calibration.

Input Modality	Precision	NPV	Brier’s Score	Overall Score
T1W	68 ± 20	43 ± 18	0.22	634
FLAIR	69 ± 20	44 ± 18	0.23	645
ADC	72 ± 18	48 ± 19	0.22	694
T1W+FLAIR+ADC	72 ± 19	46 ± 17	0.23	668
T1W *	64 ± 18	41 ± 17	0.25	664
FLAIR *	69 ± 18	47 ± 18	0.24	689
ADC *	72 ± 15	53 ±20	0.21	742
T1W+FLAIR+ADC *	68 ± 18	49 ± 18	0.23	711

**Table A6:** Complementary performance metrics of different deep learning models for predicting resistant pixels inside the GTV area. Initial models trained only on in-house cohort (N = 64). Models with * show training on the total training dataset (N = 104), including both cohorts (in-house and RHUH-GBM). All reported metrics were calculated on the same separate in-house test dataset (N = 16). NPV = negative predictive value = $\frac{\mathrm{TN}}{\mathrm{TN}+\mathrm{FN}}$ (TN = True Negatives, FN = False Negatives); overall score was defined as different weights assigned to metrics according to their importance in clinical application = Accuracy + Balanced Accuracy + ROC AUC + PR AUC + Precision + Specificity + 2 × (F1-Score + Sensitivity). Brier’s score was used to evaluate the accuracy of the probabilistic predictions, where lower scores indicate better predictive performance [29,30]. The Brier scores range from 0 to 1, where 0 is perfect calibration.

Model	Threshold	NPV	Briers Score	Overall Score	Trainable Parameters
UNet	0.59	48 ± 19	0.22	694	121 K
Pretrained ResNet34	0.57	48 ± 21	0.25	697	22 M
UNet with frozen medical VLM	0.49	46 ± 19	0.25	680	8 M
UNet *	0.47	53 ± 20	0.21	742	121 K
Pretrained ResNet34 *	0.55	52 ± 21	0.23	739	22 M
UNet with frozen medical VLM *	0.65	50 ± 19	0.24	719	8 M
UNet with BLIP image-text *	0.40	52 ± 19	0.25	735	52 M

* Clinical text information, including tumor location (temporal, frontal, basal ganglia, thalamus, 3rd ventricle), resection status (STR, GTR, biopsy), MGMT status (methylated, unmethylated), additional treatments (lomustine, radiation, avastin, retreatment RT, resection, re-resection, dose-dense TMZ), and corpus callosum involvement. STR = subtotal resection; GTR = gross total resection; MGMT = O^6^-methylguanine-DNA methyltransferase; TMZ = Temozolomide.

**Table A7:** Patient-level 3D Dice performance across the held-out test set. Patient-level 3D Dice was computed separately for each test patient by pooling true-positive, false-positive, and false-negative pixels across all retained GTV slices within that patient, rather than averaging slice-level Dice values. Patients are grouped by recurrence pattern. Dice scores were not calculated for patients with no GTV recurrence because no true resistant pixels were present. In contrast to Appendix A Table A3, which reports recurrence-pattern subgroup performance, this table reports individual patient-level Dice values to show inter-patient variability.

Anonymous Patient	Recurrence Pattern	UNet	Pretrained ResNet34	UNet with Frozen Medical VLM	UNet with BLIP Image-Text
Patient 01	No GTV recurrence	NA	NA	NA	NA
Patient 02	No GTV recurrence	NA	NA	NA	NA
Patient 03	No GTV recurrence	NA	NA	NA	NA
Patient 04	Partial recurrence	0.52	0.51	0.19	0.43
Patient 05	Partial recurrence	0.10	0.11	0.19	0.17
Patient 06	Partial recurrence	0.70	0.65	0.69	0.66
Patient 07	Entire-GTV recurrence	0.42	0.33	0.41	0.57
Patient 08	Entire-GTV recurrence	0.89	0.89	0.86	0.93
Patient 09	Entire-GTV recurrence	0.64	0.46	0.08	0.52
Patient 10	Entire-GTV recurrence	0.80	0.65	0.55	0.48
Patient 11	Entire-GTV recurrence	0.90	0.87	0.78	0.82
Patient 12	Entire-GTV recurrence	0.85	0.85	0.82	0.88
Patient 13	Entire-GTV recurrence	0.62	0.71	0.67	0.85
Patient 14	Entire-GTV recurrence	0.75	0.73	0.44	0.65
Patient 15	Entire-GTV recurrence	0.63	0.43	0.13	0.10
Patient 16	Entire-GTV recurrence	0.91	0.90	0.89	0.87
